# Dietary Micronutrients from Zygote to Senility: Updated Review of Minerals’ Role and Orchestration in Human Nutrition throughout Life Cycle with Sex Differences

**DOI:** 10.3390/nu13113740

**Published:** 2021-10-23

**Authors:** Mohamed A. Farag, Samia Hamouda, Suzan Gomaa, Aishat A. Agboluaje, Mohamad Louai M. Hariri, Shimaa Mohammad Yousof

**Affiliations:** 1 Department of Pharmacognosy, College of Pharmacy, Cairo University, Cairo 11562, Egypt; 2 Department of Chemistry, School of Sciences & Engineering, the American University in Cairo, New Cairo 11835, Egypt; Samiasalah@aucegypt.edu (S.H.); suzyaj@aucegypt.edu (S.G.); agboluajeaishat@aucegypt.edu (A.A.A.); hariri1994@aucegypt.edu (M.L.M.H.); 3 Department of Medical Physiology, Faculty of Medicine in Rabigh, King Abdulaziz University, Jeddah 21589, Saudi Arabia; 4 Department of Medical Physiology, Faculty of Medicine, Suez Canal University, Ismailia 41522, Egypt

**Keywords:** micronutrients, minerals, microelements intake, microminerals deficiency, malnutrition, life cycle

## Abstract

Micronutrients such as selenium, fluoride, zinc, iron, and manganese are minerals that are crucial for many body homeostatic processes supplied at low levels. The importance of these micronutrients starts early in the human life cycle and continues across its different stages. Several studies have emphasized the critical role of a well-balanced micronutrient intake. However, the majority of studies looked into or examined such issues in relation to a specific element or life stage, with the majority merely reporting the effect of either excess or deficiency. Herein, in this review, we will look in depth at the orchestration of the main element requirements across the human life cycle beginning from fertility and pregnancy, passing through infancy, childhood, adolescence, and reaching adulthood and senility, with insight on the interactions among them and underlying action mechanisms. Emphasis is given towards approaches to the role of the different minerals in the life cycle, associated symptoms for under- or overdoses, and typical management for each element, with future perspectives. The effect of sex is also discussed for each micronutrient for each life stage as literature suffice to highlight the different daily requirements and or effects.

## 1. Introduction

Minerals are inorganic elements essentially required by humans to carry out important functions throughout the human life, with approximately 20 mineral elements discovered to be vital for their electrolyte balance, structural and functional roles [1]. The most abundant elements are carbon, hydrogen, oxygen and nitrogen, accounting for approximately 96% of the human body weight although they are not listed as nutrient minerals, whereas macro minerals (i.e., magnesium, sodium, calcium, sulfur, chlorine, phosphorus and potassium) and micro minerals (i.e., manganese, zinc, cobalt, copper, molybdenum, fluoride, iron, iodine and selenium) make up the remaining percentage [2].

Several health benefits have been associated with micronutrients’ (minerals and vitamins) intake attributed to their functions in the enzyme system as cofactors and coenzymes, healthy bones and teeth formation, maintenance of body tissues as well as other physiological and biochemical functions [3]. These elements are derived from different dietary sources typically required by humans at different levels (generally less than 100 milligrams per day) to prevent against these deficiencies by nutrient supplementations and or fortifications [4,5,6]. Examples include salt iodization, iron and folate supplementation programs for child-bearing women, micronutrient powders and bio-fortification of crops [3].

Deficiency of trace element nutrients is more likely to be encountered in pregnant and lactating women than normal adults which can only be met through eating a balanced diet [7]. For example, hypocalcemia negatively influence the permeability of membranes and the power of smooth muscle contractions, which can impact blood pressure and could initiate preterm uterine contractions [8]. Furthermore, aging process involves cellular senescence in body tissues and organs ultimately leading to the production of reactive oxygen species (ROS) which impairs proper function of cells [9,10]. Therefore, adequate daily intake is crucial. For example, copper is essential for maintaining cognitive function in elderly people and selenium plays an important role in boosting the immune system [11,12]. Additionally, these microminerals can prevent deterioration of age-related diseases (i.e., the intravenous iron helps in heart failure patients) [13]. Consequently, monitoring nutrient intake, especially crucial minerals and vitamins, is required in geriatric preparations.

Several studies have emphasized the critical role of a well-balanced micronutrient intake. However, the majority of them looked into or examined such issue in relation to a specific element or life stage, with the majority to merely report the effect of either excess or deficiency. Herein, in this review we will cast light on the main micronutrient requirements across the human life cycle beginning from the importance for fertility and pregnancy, passing through to the infancy, childhood, adolescence, and reaching to adulthood and senility. Emphasis is given towards approaches to the different minerals role in life cycle, associated symptoms for under- or overdoses and typical management for each element. The effect of sex is discussed for each micronutrient for each life stage as literature exists to highlight for the different daily requirements and or effects.

### Search Strategy

To conduct this review an electronic search has been done through web of Science, PubMed and Google Scholar search engines. The keywords used were “micromineral, microelements, selenium, iron, cobalt, copper, fluoride, zinc + fertility, childhood/children, pregnancy, adults, elderly”. Additionally, to find out the role of sex differences and age on the recommended daily allowance, we used “selenium, iron, cobalt, copper, fluoride, zinc + required daily allowance” as keywords. Both review articles and original researches related to the topic were included. Papers were excluded for which only abstract was found, preprints and conference proceedings. Animal studies were included only in case there were no or few human studies.

## 2. Selenium

Selenium (Se) takes part in selenoproteins (selenium-dependent enzymes) which functions as redox regulator, antioxidant, in immunoglobin production, lymphocyte proliferation and as an antiviral and anticancer element inside the body [14,15,16]. In nature, selenium exists in organic (selenomethionine and selenocysteine) and inorganic (selenate and selenite) forms, with both to represent good dietary sources of selenium. Plants accumulate inorganic selenites from the soil and convert them to organic forms which suggests that the latter will most likely be the major dietary source of selenium [17,18].

Although selenium is an essential trace mineral required in small quantities, it has potential toxicity at high levels of consumption. The optimal recommended daily consumption of selenium is 55 µg, although generally, recommended dietary allowance (RDA) may differ depending on several factors such as geographical area and selenium level in the diet [19]. Recommended dietary allowances of selenium [17] is summarized in Table 1. Excess accumulation of selenium has been linked to hepatic necrosis, and cardiac muscle dystrophy [20]. In pigs, acute selenosis develops after an high dietary consumption of more than 20 mg per kg of body weight of the element selenium [21]. Chronic selenosis develops after consuming diets containing 5–20 ppm selenium for an extended period of time in pigs [21]. Selenium poisoning is characterized by symptoms such as blindness, stiffness of the bone, severe anemia and hair loss [22], more likely to exist in regions with extremely high selenium levels above average and in individuals that consume up to 5 mg per day or from atmospheric inhalation of as much as 0.2 mg/m^3^ [23]. Serum concentration of 60–120 ng/mL is the best indicator for selenium status inside the body [24].

Selenium deficiency does not always manifest with an obvious illness, although individuals with selenium deficiency often experience more physiological stress. Insufficient selenium can adversely affect enzyme activities that depend on selenium i.e., iodothyronine deiodinases, selenoprotein W and glutathione peroxidases (GPx1 and GPx3) [25].

Patients on long-term total parenteral nutrition (TPN) without selenium as part of the nutrients show symptoms such as inflammation of the heart muscle, muscle weakness and wasting [25]. Likewise, Keshan disease, a type of cardiomyopathy, is fatal and predominant in selenium-deficient individuals. Supplementation of selenium in diet has been reported to prevent development of Keshan disease although unable to reverse cardiac muscle damage. Selenium deficiency is also common in patients with Kashin–Beck disease (KBD), a condition characterized by osteoarthritis and dwarfism [26,27]. Since selenium is important in many body functions such as the immune modulatory functions, it is thus important to understand how it changes at different life stages as illustrated in the next subsections.

### 2.1. Fertility and Perinatal Period

Selenium stimulates and aids in female fertility. Nonetheless, there have been contradictory reports as to whether or not selenium supplementation is important during pregnancy [28]. Nevertheless, there is a consensus that selenium is crucial in pregnancy as growing fetus causes oxidative stress with selenium-containing compounds needed to mitigate against such stress. The fetus has some level of protection against selenium toxicity, however its deficiency can lead to low birth weight and intrauterine growth restriction [29]. Similarly, low levels of selenium decreases the activity of glutathione-GPx peroxidase that is responsible for protecting the placenta against oxidative stress [30]. High pre-pregnancy body mass index (BMI) has also been related with significantly low levels of selenium during early stage of pregnancy [31]. Hypertension in at least 1 out of 10 pregnant women especially in the second trimester has been reported to be largely attributed to selenium deficiency [29].

### 2.2. Childhood and Adolescents

There are very few reports on the significance and specific role of selenium in childhood and adolescence. However, inherent deficiency of selenium has been reported in preterm infants due to low maternal selenium levels leading to low umbilical cord levels, early gestational age and low birth weight. The lack of adequate data makes selenium deficiency in children underdiagnosed with increasing population of suspected cases [32]. Children and adolescents suffering from Hashimoto’s thyroiditis and hypothyroidism have also showed low serum levels of selenium confirming that selenoproteins are important components of the thyroid function [33].

### 2.3. Adulthood and the Elderly

Selenium is important in adults and elderly people for the stimulation of both acquired and innate immunity attributed to selenium antioxidant properties. Selenium is known to improve and enhance immunity of cells by increasing production of cytokines specifically to enhance production of interferon gamma (IFN-γ) and interleukin 10 (IL10) production which mitigates against aging-induced effects, as well as inflammatory effects in the immune system [12]. Aging affects lifespan with low serum selenium levels found to promote age-related diseases such as tumors, cardiovascular and neuropsychiatric diseases as well as aging of the skin [34]. The supplementation of selenium in adults and older people is thus important and to be achieved from consumption of selenium-fortified milk, fish and seafood.

## 3. Iodine

Iodine is a trace mineral essential in the biosynthesis of thyroid hormone and to regulate basal metabolic rate; thyroid hormones are required for normal neurodevelopment and growth especially in children [35,36]. Iodine in food exists in different chemical forms as iodide, inorganic iodine (I_2_), iodate, as well as salts of potassium and sodium. In the gastrointestinal tract, iodate is reduced and absorbed as iodide while in the duodenum and stomach, iodide is rapidly absorbed and circulates into the thyroid gland with the remainder excreted in the urine [37].

Iodine deficiency disorder (IDD) has become a public health problem affecting people of all age groups especially children and breastfeeding mothers [38]. Under normal circumstance, the thyroid gland of a healthy individual is expected to encompass 70–80% of the total iodine in the body, which is about 15–20 mg while the body uses about 80 µg to produce thyroid hormones In the case of deficiency, the hypothalamus–pituitary–thyroid pathway becomes activated such that the volume of the thyroid gland increases until goiter becomes obvious, to be managed with surgery and replacement of thyroid hormone [35]. However in a patient with chronic iodine deficiency, iodine level in the thyroid can be as low as 1 mg leading to a spectrum of IDD which includes hypothyroidism, goiter, mental retardation and other growth abnormalities [39,40]. Recommended dietary intake of iodine [41] across the human life is summarized in Table 1.

As with most micronutrients, the daily requirements for iodine changes at different life stages, generally ranging between 90 µg to 290 µg. Daily iodine consumption below 50 µg may cause deficiency, while more than 350 µg may lead to excess iodine and above 600 µg can cause damage to the thyroid function [35] suggestive for the value of RDA levels in order to avoid deficiencies and potential toxicity.

### 3.1. Fertility and Perinatal Period

Iodine requirements increases during pregnancy and lactation. Deficiency in pregnancy can lead to maternal and fetal hypothyroidism, and affect fetus neurological development which can become more severe as deficiency progresses for a longer time without treatment. Supplementation especially with salt iodization has been reported to be the most cost effective program in improving iodine status as it has been shown to increase development of children by up to 20%. Iodine supplementation in pregnant women have shown reduced cases of perinatal and infant mortality, low birth weight and cretinism [42].

### 3.2. Childhood and Adolescence

While the effect of mild maternal iodine deficiency is not clear to affect offspring development, albeit chronic deficiency has been associated with increased risk of cognitive disability in infants. In addition, severe childhood disability adversely affects somatic growth, though early detection and correction of deficiency through supplementation improves both cognitive and motor functions in children [43]. It is suggested that the central nervous system (CNS) development may be sensitive to thyroid hormone deficiency because the hormone receptors are present in the brain of the fetus as early as nine weeks old. In cases of severe maternal iodine deficiency, thyroid hormone production becomes compromised and eventually leads to fetal hypothyroidism and impaired brain development [44]. The most reliable indicator for iodine deficiency in childhood is through the thyroid-stimulating hormone in newborns and thyroglobulin in older children [43].

### 3.3. Adulthood and the Elderly

In adults, iodine deficiency causes low productivity at work, impaired mental functions, toxic nodular goiter and susceptibility to nuclear radiation and development of thyroid cancer. In most of the elderly population above 55 years, hyperthyroidism (excess production of thyroid hormone) is most common [45,46]. Adult deficiency could be related to decreased milk consumption [47]. It is probable that adults may show greater chances of developing iodine deficiency when compared to the younger population due to their lower milk consumption [48]. The decreased milk consumption may be linked to some factors in the elderly population, such as dietary habits, avoiding milk for its cholesterol level, reduced appetite, gustatory dysfunction, reduced basal metabolism, and economic issues [49].

## 4. Iron

**Figure 1:**
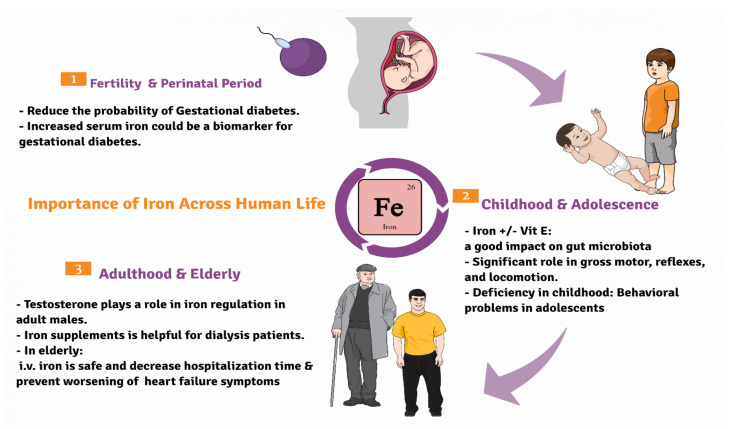
Diagrammatic sketch showing the importance of iron across life cycle of humans beginning from the fertile stage, childhood to the elderly.

Iron is an essential micromineral involved in many crucial body functions, Figure 1. It is found in diets mainly in meat, legumes and leafy vegetables. Primarily, most of the body iron is present in the form of hemoglobin in red blood corpuscles, where it plays a major role in oxygen transfer process [50]. Overall, adult females typically require more iron than men attributed to the fact that adult females loose blood during menstrual cycle [51]. Recommended dietary allowances [52] of iron throughout life is illustrated in Table 2.

### 4.1. Fertility and Perinatal Period

Several studies have related iron intake at this stage to the prevention of illnesses during pregnancy and beyond. Others have tested the effect of iron, iron and folic acid and a dietary supplement containing similar amounts of iron and folic acid on preconception women in comparison to a control group receiving only folic acid regarding anemia and iron stores. After pregnancy and delivery, blood analysis revealed that anemic status did not change. However, iron stores as indicated by ferritin levels were found higher with supplementation with their babies to show higher ferritin levels than those in folic acid alone [53].

Interestingly, investigators have retrospectively examined the association between the preconception consumption of heme iron, non heme iron and iron supplementation on gestational diabetes mellitus revealing that non-heme iron supplementation reduce the probability of gestational diabetes [54]. On the other hand, an earlier study has reported that increase in serum iron increases at early pregnancy and to likely lead to gestational diabetes. The study further suggested that serum iron of 100 μg/dL or higher could be used as a marker to diagnose diabetes during pregnancy [55]. These findings were supported by another study that showed the effect of iron supplementation on pregnant women with high risk of diabetes during pregnancy suggestive that participant consuming higher amounts of iron) median daily intake: 27.1 mg) are at a greater risk. The mechanisms behind that are though still not fully understood. However, this can be explained by enhanced oxidative stress and damage of pancreatic beta cells or diminished metabolism of insulin by liver leading to peripheral hyperinsulinemia by iron overload [56]. However, it should be noted that no control healthy group was included in this study being a survey without giving a controlled dose of iron supplementation.

Concerning iron homeostasis during pregnancy, hepcidin hormone is responsible for regulating absorption and mobilization of iron. A clinical study revealed elevated hepcidin levels in pregnant women in the first trimester compared to non-pregnant women, with later drop to remain constant during the rest trimesters. Additionally, this study investigated the relationship between iron and iron-related biomarkers, and spontaneous abortion revealing that this group of aborted females had higher iron, transferrin and serum ferritin levels than the healthy pregnant group. These results suggest that either pregnancy causes disturbance in iron regulation or iron dysregulation cause abnormalities in pregnancy [57].

To assess iron status after delivery, lactating women receiving iron supplementation were measured for their iron status as well as oxidative stress markers, with improved iron levels compared to control. However, no significant change was observed in oxidative stress markers [58]. In addition, iron supplementation to lactating women increased levels of this microelement in breast milk [59].

### 4.2. Childhood and Adolescence

Iron is an important micronutrient at this early stage, yet its level in breast milk is very low (0.4 mg/L) [60]. Consequently, many infant formula that are fortified with iron among other micronutrients are typically administered with several studies conducted to compare between infants on iron-fortified diet and those on iron-poor diet in regards to their several physiological, pathological and psychological aspects. Some investigators have concluded that iron supplementation through either exclusively iron-fortified cereals, iron- and zinc-fortified cereals or pureed meat for infants enhanced iron profile avoiding anemia, concurrent with stimulation of gut microbiota [61]. All three groups showed increased serum hemoglobin and serum ferritin levels. Nevertheless, the absence of a control group hinders from assessing whether these changes occurred solely due to administered diet or not, as the study did not consider the baby’s normal development. Another study has reported that addition of vitamin E to iron showed a positive impact on gut microbiota than iron alone, though with no change in iron profile upon the addition of vitamin E, nor in inflammatory markers [62]. In addition, iron appears to have a significant effect on motor development in infants compared to control and likewise in supplemented babies showing significantly, better scores in terms of overall gross motor, reflexes, and locomotion [63]. Iron deficiency has been linked in many studies to attention deficits and hyperactive disorder (ADHD) in childhood and adolescence [64,65]. Nevertheless, further studies are to be conducted to examine the effectiveness of iron supplementations in ADHD. Additionally, further studies to investigate the role of brain iron as a biomarker for ADHD are needed [65].

### 4.3. Adulthood and the Elderly

It is well known that testosterone plays a major role in iron regulation in adult males, with men suffering from hypogonadotropic hypogonadism showing decreased serum iron levels. Hypogonadal males have a five-fold increased risk to be anemic compared to eugonadal counterparts [66]. To establish the mechanism through which testosterone contributes to iron homeostasis, Dhindsa et al. (2016) assessed certain iron-related biomarkers before and after hormonal therapy for males with hypogonadotropic/hypogonadism revealing that there was a positive relation between testosterone and erythropoietin, as well as the gene expression of ferroportin versus negative relationship with hepcidin [67]. To confirm whether testosterone itself is the main male hormone responsible for iron metabolism or its metabolite dihydrotestosterone.A study on elderly males has been conducted to administer either testosterone with or without tesfinasteride, drug that hinders the formation of dihydrotestosterone. Results showed no significant difference regarding androgen-mediated blood formation or iron regulation suggestive that the effect is principally-mediated by testosterone [68]. On the other hand, hemochromatosis is a hereditary disease that can occur due to iron overload. [69].

Heart diseases are common in adults and elderly people, with intravenous administration of iron found to be safe and useful in decreasing hospitalization and deterioration of heart failure symptoms [13]. The majority of research have utilized ferric carboxymaltose (maximum dose of 200 mg per setting) or I.V. iron sucrose (maximum dose of 1000 mg per week) [70]. In addition to cardiac diseases, acute or chronic renal failure patients usually suffer from anemia due to the reduced amount of erythropoietin concurrent with increase in hepcidin that blocks iron intestinal absorption [71]. Therefore, iron and erythropoietin are usually administered to patients on dialysis. A group of researchers introduced iron to patients as ferric citrate which not only improved iron status, but rather served as a phosphate binder to avoid against hyperphosphatemia that occur in chronic kidney diseases [72].

## 5. Cobalt

Cobalt (Co) is a relatively rare metal in the Earth’s crust that mammals require in the form of cobalamin (vitamin B12). Cobalt is found in the human body at small levels, with 85% in the form of vitamin B12 primarily derived from drinking or eating Co containing foods [73]. Cobalt, as one of the essential minerals for human health, possesses a key function in several biochemical activities such as biosynthesis of nucleic acids and amino acids, as well as erythrocytes production [74]. Nowadays, lots of cobalt (Co)-containing dietary supplements are sold on the market, and many producers have suggested a daily dose up to 1 mg to aid in metabolizing fat and carbohydrates, protein and red blood cell formation [75]. Cobalt enters the body through several routes, including food, the respiratory system, the skin, and biomaterials. Recommended dietary allowances [76] of cobalt across life cycle are listed in Table 2.

### 5.1. Fertility and Perinatal Period

Almost 50% of male infertility causes are not clear, with micronutrients known to exert an effect on spermatozoa, sperm quality, and infertility in men. Co concentration and its effect on the quality of sperm, was evaluated in human semen. Results revealed that males with normal sperm motility had lower Co levels than males with asthenozoospermia, or reduced sperm motility. These findings also indicate that measurement of Co levels in seminal plasma would be a beneficial diagnostic means among others for infertility in men [77].

Until now, there have not been enough studies to correlate between serum levels of Co during pregnancy and preterm birth (PTB) risk, which is a birth before 37 weeks of gestation. Nevertheless, Co deficiency has been linked to problems in vitamin B12 biosynthesis likely to increase predisposal to developmental abnormalities and failure in infants. A study on human females that assessed the relationship between Co levels of both maternal and umbilical cord and the risk of PTB suggested that the low levels of maternal and umbilical cord serum cobalt increased the risk of PTB [78].

### 5.2. Childhood and Adolescence

Co has a significant effect on the stimulation of erythropoietin and haemoglobin production. Elevated levels of Co have been detected in blood at decreased body iron stores. A study was performed to assess the levels of micronutrients, including Co, and their deficiencies in children aged 0 to 3 years with iron deficiency anaemia (IDA) revealing lower Co levels compared to control groups. Co is known to stimulate erythropoietin production through the activation of the transcription factor hypoxia-inducible factor 1α (HIF-1α), and affect the synthesis of DNA via the development of stem cells and synthesis of haemoglobin. This suggests that Co deficiency is among the factors that cause or can lead to IDA in children [79].

### 5.3. Adulthood and the Elderly

Lately, there has been increase in the consumption of products that might contain increased levels of essential elements such as supplements that contain Co marketed as energy enhancers. Nevertheless, there is not enough data concerning Co body burden and blood levels following the consumption of such products. Increase in Co serum level was observed in four healthy adult males after the consumption of a Co supplement (0.4 mg Co/day) for 15 or 16 days to increase from 0.5 µg/L to reach 1.8 to 5.1 µg Co/L. More research studies on Co blood levels after long consumption of Co supplements would provide beneficial data for the assessment of the health status adults who consume Co for the long term [75].

## 6. Fluoride

Water, pharmaceuticals, herbicides, insecticides, fertilizer residues, dental restorative materials, dental goods (toothpastes and mouth rinses), pediatric supplements, beverages prepared with fluoridated water, and food are all sources of sodium fluoride (NaF) [80]. The environmental protection agency (EPA) recommends acceptable fluoride levels in drinking water to be around 4.0 mg/L, with majority of bottled water fluoridation at 1.5 mg/L according to the world health organization (WHO) [81]. Fluoride deficiency is known to lead to dental caries and can be overcome by water fluorination. However, concerns regarding the unsafety and toxicity due to the over use of fluoride rendered many countries to prohibit fluorination [82]. Recommended dietary allowances [83] of fluoride are displayed in Table 3.

### 6.1. Fertility and Perinatal Period

An animal study has documented that fluoride impairs male rabbits’ reproductive functions, with such effect related to fluoride exposure time [80]. In that study, exposure to NaF- and F-contaminated groundwater resulted in a significant reduction in total sperm count, sperm motility, concentration of serum testosterone compared to control in a dose dependent manner and reversible on short-term exposure [84]. Negative effects were also reported in female mice, confirming the negative effects of excess fluoride on fertility. In that study, adult female mice were given tap water containing different doses of sodium fluoride (NaF); (0, 100, 200, and 300 ppm NaF) for 4 or 12 weeks. The number of pregnant mice, implantations, viable fetuses, and resorptions have been used to determine the effect of NaF exposure on fertility. Mice exposed to 300 ppm showed a considerable increase in the relative ovary weights and a diminished embryo weights. On the other hand, at all concentrations tested, 12 weeks of exposure to NaF resulted in a considerable drop in pregnancies, with mice exposed to 200 and 300 ppm showing significantly higher relative ovary weights and fewer viable babies [85]. More clinical evidence is needed in humans to be conclusive regarding fluoride negative impact on fertility at high doses.

For pregnant women in Northern California, community water fluoridation is still a primary source of fluoride exposure [86]. It has been reported that lower intelligence quotient (IQ) scores in children aged 3 to 4 years were linked to mother exposure to higher fluoride levels during pregnancy, and to suggest that fluoride intake during pregnancy ought to be reduced. A 3.66 lower IQ score was related with a 1 mg higher daily fluoride consumption among pregnant mothers in both sexes [87]. Another study has documented that increased water fluoride above the recommended level has been associated with anemia in 402 patients (67%), with hemoglobin values ranging from 6.2 to 11.9 g/dL. Patients (13.5%) had negative fetal outcomes, including abortion and congenital malformations [88].

### 6.2. Childhood and Adolescence

Dental and skeletal fluorosis have been found to be common among children in India, with 15 dental fluorosis affected 41 (89%) of the 46 children, while skeletal fluorosis affected 18 (39%) of the children [89]. Fluoride levels in water ranged from 0.5 to 12.6 parts per million. Furthermore, signs of oxidative stress were observed in eight children with endemic skeletal fluorosis manifested by increased malondialdehyde levels, reduced glutathione and uric acid levels, as well as an increase in glutathione activity [89]. A study assessed the relationship between contemporaneous urine fluoride levels and physical markers of pubertal development in children using data from a Mexican birth cohort study. In males, an increase in urine fluoride was linked to a delay in pubic hair growth and genital development. Whilst, in females, no significant connections was observed [90]. A cross-sectional research from the National Health and Nutrition Examination Survey (NHANES) correlated fluoride exposure and sleep patterns among older adolescents (about 17 years of age) in the United States in 2015–2016. There was a delayed bedtime and waking time as well as enhanced odds for symptoms of obstructive sleep apnea. An interquartile range rise in water fluoride was linked to a 1.97-fold increase in the likelihood of experiencing sleep apnea symptoms [91]. Based on the updated guidelines of European Academy of Pediatric Dentistry Regardless of whether the program is community-based or individual-based, the usage of fluoride must be regulated between the prediction of caries prevention and the potential dangers of fluoride-related side effects [92].

### 6.3. Adulthood and the Elderly

Fluoride is the most helpful in preventing dental cavities among minerals [93], with fluoride levels in saliva and plaque aid to prevent and reverse caries by preventing demineralization and increasing remineralization. It has been well documented that fluoride “supplements” are best employed as a topical administration mechanism by sucking or chewing tablets or lozenges to prevent dental caries [92]. For adults at an elevated risk of caries, prescription toothpastes containing more than 1500 ppm F (typically in the range of 2000 to 5000 ppm F) are recommended [94]. A systematic review and meta-analysis was conducted to assess fluoride efficacy in caries prevention in adults in 2007. For all adults, aged 20+ and 40+ years, it was evident that fluoride appears to prevent caries in adults of all ages [95].

In the elderly, hyposalivation and tooth root exposure are issues that necessitate particular oral care. The properties of specific toothpastes are thus very important in that context [96]. When compared to regular F toothpaste (1000–1450 ppm F), there is reasonable evidence that high fluoride dentifrices (2500–2800 and 5000 ppm F) considerably boost fluoride level in saliva during the day and in plaque in elderly patients. Moreover, using toothpaste containing 5000 ppm F reduces plaque accumulation, lowers the incidence of mutant streptococci and lactobacilli, and may encourage calcium fluoride deposits to a greater extent than using typical toothpaste [97].

## 7. Zinc

Zinc is the most abundant micronutrient in the human body after iron and one of the chief trace elements in the human body detected at approximately 1.4–2.3 g Zn in the body of an adult. High levels of zinc are present in all body tissues at highest level (85%) in muscles and bones, followed by prostate and the eye. It catalyses the activity of several enzymes involved in both protein structure folding and gene expression regulation. Zinc is also needed structurally for of zinc-containing proteins, namely zinc finger proteins (ZFP), biggest superfamily of nucleic acid-binding proteins. It is also fundamental for cell growth, differentiation and homeostasis, asides from its unique role in connective tissue growth and maintenance and immune system integrity [98]. For recommended dietary allowance of zinc [99], refer to Table 3.

### 7.1. Fertility and Pregnancy

Mammalian semen has a high level of total zinc owing to its critical role in spermatogenesis, germination, quality of sperm and fertilization. In males, zinc has significant characteristics such as its antioxidant nature, with its deficiency related to hypogonadism, sperm abnormalities and inadequate evolution of secondary sex characteristics [100]. Seminal zinc is also known to affect head–tail attachment/detachment and nuclear chromatin condensation/decondensation. Men who are smokers and infertile exhibit elevated reactive oxygen species, which affects zinc level in seminal plasma to an extent that affect spermatozoa. Zinc is significant for the maintenance of reproductive organs lining [101,102] posing it as potential element in sustaining male fertility.

Likewise, in females, pregnant women from Ethiopia in their first 8 weeks of pregnancy were assessed for serum zinc levels, with 36.7% showing low levels, concurrent with 13.4% underweight among 329 new-borns. The findings suggested that serum zinc was positively correlated with birth weight, though more intervention studies are needed to further prove the findings in other races and age groups [103].

### 7.2. Childhood and Adolescence

Zinc is a major micronutrient in the process of growth and for the immune system functions. In developing countries, children are more prone to zinc deficiency due to the lack of adequate nutrition asides from loss of zinc through excretion as a result of periodic diarrhea [104,105]. A study on the effect of zinc on improving pneumonia recovery showed positive response with decreased hospitalization time [106,107]. In contrast, some trials reported the insignificance of zinc supplementation on shortening the period of pneumonia resolution [108,109]. Urinary tract infection (UTI) is a prevalent disease among children regarded as the most common infection after common colds, with evidence suggesting that nutrients deficiency might be among the causes of UTI in children. Many studies have evaluated its role in infectious diseases, with one to show a strong association between zinc deficiency and high risk of UTI in children [110], and has yet to be confirmed in other infections to be conclusive.

### 7.3. Adulthood and the Elderly

Globally, kidney stones are prevalent among adults with calcium stones the most common type. There is also 50% chance of kidney stone recurrence risk post-surgery. Trace elements especially zinc, have been associated with kidney stone formation, although this is still controversial, as different studies have reported that dietary zinc intake is not even related to kidney stones. Discrepancy might be attributed to that zinc at low levels could inhibit the crystal growth, while higher doses might boost the production of amorphous calcium phosphate [111,112].

The COVID-19 infection causes serious intense acute respiratory disorder coronavirus 2 (SARS-CoV-2), mainly infecting older adults and people aged >80 years [113,114]. Due to the lack of therapy for this virus, it is necessary to reach other methods that can aid in disease control. Determination of zinc level and COVID-19 infection revealed that Zn anti-inflammatory properties might contribute to the immune function improvement and decreasing the infection risk. This could suggest that zinc supplementation in the elderly could be helpful in reducing such currently challenging pandemic [115].

Metabolic syndrome (MetS) is considered a cluster of cardiovascular risk factors, with high frequency worldwide owing to new dietary habits. Effect of Zn level on MetS is contradictory. Some studies revealed that there is no relationship between plasma zinc levels and MetS, whereas others reported that elevated levels of serum zinc is associated with MetS in adults [116,117]. A cross-sectional investigation of zinc levels that was performed on 1111 adult men and 1290 adult women, with MetS prevalence as 39.6% and 28.1% in men and women, respectively, suggested for a gender difference between association of serum zinc levels and MetS. The results showed that serum zinc was positively associated with MetS in men, while negatively associated in women having a protective effect against MetS [118].

## 8. Manganese (Mn)

Manganese is a mineral that is required for intracellular activity. It is an enzyme activator and a component of various enzymes, including arginase, glutamine synthases (GS), pyruvate carboxylase, and Mn superoxide dismutase. Mn is required for development, digestion, reproduction, antioxidant defense, energy synthesis, immunological response, and neural activity modulation [119,120].

Dietary manganese does not cause any adverse health effects and has no risk at the usual intake levels with an estimated adequate intake (AI) level of 2.3 mg/day. However, excess Mn tends to accumulate in the liver, pancreas, bone, kidney and brain causing hepatic cirrhosis, polycythemia, and Parkinsonism-like symptoms. Mn deficiency is rare, but if it occurs it is associated with symptoms such as impaired growth, skeletal abnormalities, depressed reproductive function, ataxia of the newborn and faults in lipid and carbohydrate metabolism [121]. Regarding the recommended dietary allowances of manganese throughout life [122], refer to Table 4.

### 8.1. Fertility and Perinatal Period

Manganese plays an important role in female reproduction and fetal development. Both excess and deficiency are associated with female infertility and adverse effects on pregnancy. Pregnant women are characterized by a significant increase in serum Mn levels by 40% compared to non-pregnant women [7]. During pregnancy, manganese aids in sustaining proper fetal development and other important aspects of metabolism. Blood Mn naturally rises during pregnancy due to mobilization from tissues and likely to contribute to pregnancy and postpartum depressive symptoms. A recent cross-sectional study of Japanese women demonstrated higher manganese intake to be independently associated with a lower prevalence of depressive symptoms during pregnancy [123]. In a birth cohort study, the association of maternal blood Mn level during pregnancy with infant birth weight and dimensions in 16,473 (8484 males and 7989 females) mother–infant pairs have been examined. Results revealed that birth weight increased up to a blood Mn level of 18.6 µg/L, and no association of blood Mn level with birth weight among female infants. The results indicate that Mn lower maternal blood levels during pregnancy or a high one during the 3rd trimester is associated with a lower birth weight, in addition to fetal intrauterine growth retardation [124].

### 8.2. Childhood and Adolescence

Mn deficiency is highly unlikely with exclusive breast milk or infant formula feeding; however, tolerable daily intake levels for Mn may be exceeded when using formula products. Regulations regarding Mn content in infant formulas have not been updated in accordance with recent research findings on Mn toxicity and often there are no regulations for young child nutritional beverages. Mn at high level in drinking water was associated with lower intelligence quotients and greater incidence of learning disabilities [7].

The essential need for Mn is reflected in national and international infant formula and food policies. In infants (4–6 months) of age, breast milk or infant formula constitutes the main source of nutrition. Breast milk contains typically 2–6 μg/L of Mn [119]. In a study of 2–16 week infants, retention of Mn was higher for formula-fed infants than for breast-fed infants due to the higher Mn level in formula. Mn absorption rates are higher in neonates, 16–37%, compared to 3% in adults making neonates more susceptible to Mn toxicity also due to diminished biliary excretion, which is the major route of Mn excretion in humans [125].

Cow milk generally has a much lower Mn level (36 μg/100 kcal) than soy, rice, or chocolate. [126]. According to Food and Drug Administration (FDA) and United States nutritional standards for manganese in infant formula, the minimum level in prepared infant formula is 5 μg/100 kcal, with no maximum level specified [127].

Concerns have recently been raised about relatively high Mn exposures and possible associated adverse effects on child neuro development. Children exposed to higher levels of Mn compared to other children have been found to have impaired cognitive development, lower IQ or intelligence scores, impaired motor and olfactory functions, atypical brain structure or function, and relatively high Mn exposures are suspected of increasing the risk of attention deficits. A benchmark dose for Mn in drinking water associated with decreased IQ has recently been calculated for school-aged children [128].

### 8.3. Adulthood and the Elderly

Manganese levels in blood among subjects ranges between 4 and 15 µg L. Manganese excess in plasma/serum is generally followed by rapid clearance through either distribution to other tissues or hepatobiliary elimination. Regarding manganese levels in whole blood, a gender effect was observed, with women generally having higher levels than men do. Additionally, it has been clearly demonstrated that iron deficiency is generally associated with increased manganese levels. Therefore, the commonly observed sex differences in whole blood manganese levels might be explained at least partially by lower iron levels generally present in women [121].

A study on the elderly examined associations between dietary manganese intake with circulating biomarkers of inflammation in 633 participants [129], suggested for positive relationship with levels of three circulating inflammatory markers (IL-1β, IL-6, and IL-8) that was statistically significant likely mediated via changes in DNA methylation. Consequently, estimated dietary intakes of manganese at levels slightly above nutritional adequacy can contribute to inflammatory biomarker production [130]

## 9. Copper (Cu)

**Figure 2:**
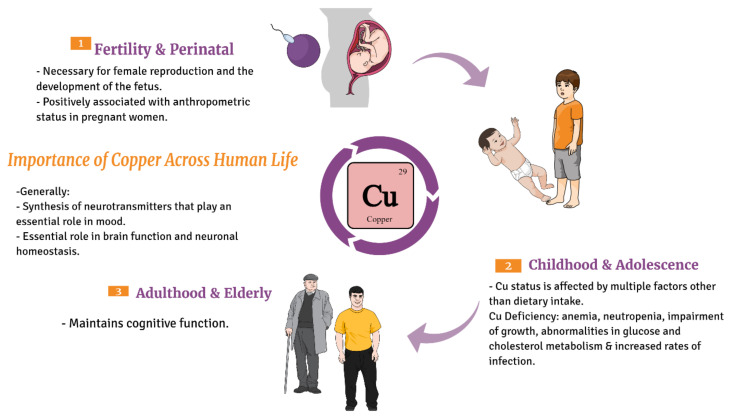
Diagrammatic sketch showing the importance of copper across life cycle of humans beginning from the fertile stage, childhood to the elderly.

Copper is essential for the survival of humans; Figure 2. It is involved in many biochemical processes supporting life, such as antioxidant defense, mitochondrial respiration, development of connective tissue, melanin biosynthesis, iron homeostasis, and peptide hormone processing [131]. It acts as cofactor in tyrosine hydroxylase and dopamine hydroxylase enzymes, which participate in the synthesis of neurotransmitters that play an essential role in mood. Ions such as copper plays an essential role in brain function and neuronal homeostasis, and long-term imbalance of these metals has been linked to neurodegeneration and neurological disorder [132]. Regarding the recommended dietary allowance [133] of copper throughout life, see Table 4.

### 9.1. Fertility and Perinatal Period

Copper is necessary for female reproduction and the development of the fetus. Excess and or lack of these elements might lead to female infertility and negative pregnancy outcomes. Compared to non-pregnant women, pregnant women have a significant 40% increase in serum Cu levels with major alterations of copper metabolism during pregnancy [7]. A previous study has documented that pregnant women supplemented with copper showed a 75% and 90% reduction in depression and anxiety symptoms during the second and third trimesters, respectively. Additionally, in the control group, the rate of infection during pregnancy was substantially higher [134]. Another study has reported a relation between severe preeclampsia and ceruloplasmin, and copper level. Women with severe preeclampsia had considerably greater Cu and ceruloplasmin levels than patients with mild (81.2 µg/dL) and severe preeclampsia (160.2 µg/dL) [135].

### 9.2. Childhood and Adolescence

Copper is transferred from the mother to the fetus, and to accumulate in the fetus mainly at the end of the gestation period mostly retained in the fetus liver to aid in preventing copper deficiency during the early months of life. After birth, liver Cu level decreases concurrent with increase in serum Cu and ceruloplasmin levels [136]. Cu is an essential micronutrient but its deficiency is rare, it has been reported in preterm infants, in infants fed with cow’s milk, and in infants recovering from malnutrition accompanied by diarrhea. Deficiency of Cu can lead to anemia, neutropenia, impairment of growth, abnormalities in glucose and cholesterol metabolism, and increased rates of infection [137].

The average Cu intake in children is at 0.80–1.90 mg/day, with infants (0–0.50 years) often to manifest a low intake of Cu (0.08–0.16 mg/day), because of its low levels in breast milk. Despite declining Cu levels in breast milk during lactation, serum Cu levels in infants are increased suggesting that Cu requirements of infants are met. Cu in breast milk appears to be well absorbed and its levels in breast milk are independent of maternal status. Cu serum level was not correlated with the daily intake in infants and in mothers suggesting that Cu status is affected by multiple factors other than dietary intake [138].

### 9.3. Adulthood and the Elderly

The estimated safe and adequate daily Cu intake recommended by the Food and Nutrition Board for adults is 1.50–3.00 mg/day. The relationship between copper and zinc and copper/zinc ratios and mood disorders, symptoms of depression in older Australian adults revealed that high Cu level, as well as Cu/Zn ratios were associated with lower depressive symptoms. Moreover, plasma copper was positively associated with brain-derived neurotropic factor (BDNF) concentration, indicating that higher copper levels were associated with lower psychological distress. The balance between copper and zinc has been positively associated with disability and mortality in the elderly. Aside from serving as nutritional or psychological indicator, Cu/Zn ratio is mostly associated with oxidative stress and inflammatory response, all to suggest copper role in maintaining brain health by acting as a cofactor for neurotransmitter synthesis [11,139]. Another meta-analysis showed conflicting results, as it revealed that high level of copper could be used as a biomarker for depressive symptoms [140]. The relation between copper and zinc concentrations rather than each element level can explain such discrepancy per se. Therefore, the Cu/Zn ratio could be a better indicator for depressive disorders than measuring each element separately [141].

Likewise, in older adult women, a high copper plasma level (>215 µg/dL) was associated with poorer cognitive function than a low plasma copper concentration (<90 µg/dL). In men, both very low and very high copper levels were associated with poor cognitive performance on tests of long-term memory, calculation, and visuomotor attention, compared to intermediate circulating copper levels. This study emphasizes the notion that copper is indeed essential for the synthesis of neurotransmitters important for maintenance of cognitive functions [11]. Elevated serum copper is associated with increased risk of mortality along with decreased serum transthyretin (TTR) in the elderly. TTR, is known as a sensitive indicator of inflammation and malnutrition [142,143].

## 10. Molybdenum

Molybdenum is a trace element that is abundant in many animal and plant dietary sources with legumes having the highest amount reaching up to 87 mg per 100 g wet weight [144,145]. Regarding its functions for humans, it is involved mainly as a cofactor in enzymes that metabolizes few chemicals such as purine, sulfur-containing amino acids, N-heterocyclic and N-hydroxylated compounds [145,146,147]. Like other nutrients, the WHO sets a daily requirement of molybdenum for adults at 100 to 300 µg/day [148]. Due to its distribution in a wide range of food materials, its deficiency is extremely rare and related mainly to a genetic disorder, termed as molybdenum cofactor deficiency, in which molybdenum fails to form its cofactor and consequently the linked enzymes fail to do their functions leading to the accumulation of undesirable substances [145,149]. In addition, its toxicity is also rare since the urinary system is sensitive towards increasing levels of molybdenum where an increase in its excretion takes place if its levels are elevated [145,150].

### 10.1. Fertility and Perinatal Period

The available studies on molybdenum during pregnancy is scarce. A prospective cohort study in Mexico found no relation between the amounts of molybdenum in the urine of pregnant women who indicated molybdenum exposure and mental retardation in the infants [151]. On the other hand, another prospective cohort study concluded that low serum levels of molybdenum in pregnant women could be associated with elevated blood glucose levels and may increase the risk of gestational diabetes [152]. However, the previous studies did not involve supplementing the pregnant women with an accurate dose of the element. Instead, the serum and urine levels originated from a regular diet.

### 10.2. Childhood and Adolescence

There are no studies that linked molybdenum intake to any disorders in infants, children and adolescents most likely because of its availability in food and rapid urinary excretion if accumulated. However, a rare genetic defect causes molybdenum cofactor deficiency, which hinders the functions of the enzymes linked to it, as described earlier. This disease is manifested by seizures at early age and the formation of xanthine stones due to the malfunction of its metabolizing enzymes. Although the mortality rates are high, some case reports suggested that through diet and symptomatic treatment, life expectancy could be prolonged. However, it is to be noted that this rare genetic disease is not related to molybdenum intake [153,154]

### 10.3. Adulthood and the Elderly

A study conducted in the United States suggested that there could be a positive relationship between the amount some metals including Mo excreted in urine and diabetes possibly through damaging the beta cells [155]. However, further investigations are still required. While numerous animal studies linked between Mo and many disorders such as sexual dysfunction [156,157], few studies are available on humans. Interestingly, one study on humans found that the increase in blood Mo was found to be related to a decrease in blood testosterone [158]. Nevertheless, these findings require further investigations. On the other hand, no studies were found regarding the effect of Mo disturbances to any diseases in the elderly.

## 11. Conclusions and Future Perspectives

**Figure 3:**
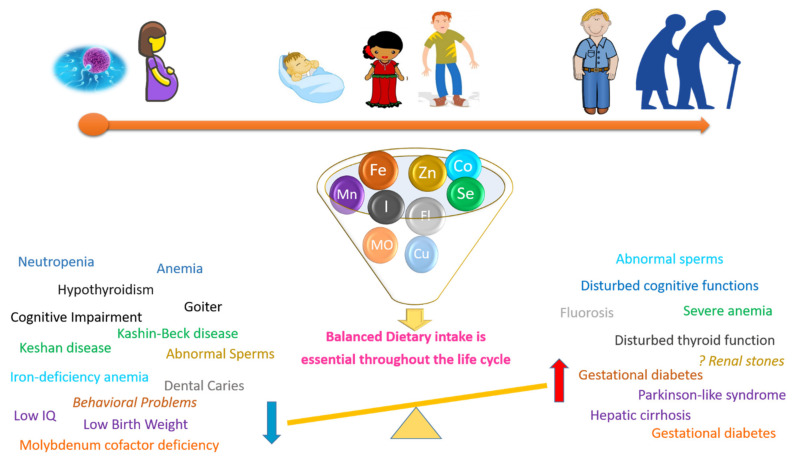
Diagrammatic sketch showing the importance of adequate balanced dietary intake of the microelements for human health across life cycle. The disturbances in the micronutrient intake either by excess or deficient intake, could lead to disturbed body homeostatic functions and diseases. Each micro mineral is coded by a color code. The right side of the figure shows the effects of excess use of the different elements. Whilst the left side shows the effects of the deficiency of the same elements. There are controversial findings regarding the role of zinc in the occurrence or renal stones. *IQ* = *Intelligence Quotient*.

This review provides a comprehensive overview of microminerals’ role and orchestration in human nutrition throughout the life cycle with sex differences highlighted as reported in literature. Additionally, we shed light on the importance of the balanced intake of these microminerals to avoid a negative outcome upon deficient or over intake of these microminerals as summarized in Figure 3. In summary, despite supplementation efforts, inadequate consumption of trace elements like iodine are fast becoming public health problems worldwide causing a strain to human performance and even economic productivity. Regarding selenium, though being well recognized for its role in immune function and reproduction, there have not been enough studies to assess its effect in key life stages such as lactation and infancy. Attention should be paid to selenium, especially, given that deficiency symptoms are not obvious. Iron plays a major role throughout life cycle not only in maintaining homeostasis, but also preventing the unwanted sequels of some illnesses like chronic heart failure in the elderly etc. Most reports related to cobalt deficiency have related it to vitamin B12 deficiency, with still not enough studies regarding its role in different life stages, as well as insufficient clear data regarding the consequences of its deficiency that is not related to vitamin B12. In contrast, the role of zinc and copper in different stages of the life cycle have been investigated considerably throughout the literature. However, there is still some controversy regarding the role of zinc in the occurrence of renal stones. Studies related to fluoride deficiency effects on fertility are still to be elucidated at clinical stages.

## Figures and Tables

**Table 1:** Recommended dietary allowance for selenium and iodine.

	Selenium			Iodine		
Life Stage	Age	Males (µg/day)	Females (µg/day)	Age	Males (µg/day)	Females (µg/day)
Infants	0–6 months	15	15	0–6 months	110	110
	7–12 months	20	20	7–12 months	130	130
Children	1–3 years	20	20	1–8 years	90	90
	4–8 years	30	30	9–13 years	120	120
	9–13 years	40	40	14–18 years	150	150
Adolescents	14–18 years	55	55	≥19 years	150	150
Adults	19–50 years	55	55	-	-	-
Pregnant	-	-	60	-		220
Breastfeeding	-	-	70	-	-	290

Sources: Khayat et al. 2017 [8]; National Institute of Health, 2021; National Institute of Health, 2021.

**Table 2:** Recommended dietary allowance for iron and vitamin B12.

	Iron			Vit. B12 (Cobalt)		
Life Stage	Age in Years	Males (mg/day)	Females (mg/day)	Age in Years	Males (µg/day)	Females (µg/day)
Children	1–3	9	9	1–3	0.9	0.9
	4–8	10	10	4–8	1.2	1.2
	9–13	8	8	9–13 years	1.8	1.8
Adolescents	14–18	11	15	14–18 years	2.4	2.4
Adults	19–50	8	18	19 years	2.4	2.4
	51–>70	8	8	-	-	-
Pregnant	19–50	-	27			2.6
Breastfeeding	19–50	-	9			2.8

Sources: Khayat et al. 2017 [8]; National Institute of Health, 2021; National Institute of Health, 2021.

**Table 3:** Recommended dietary allowance for fluoride and zinc.

	Fluoride			Zinc		
Life Stage	Age in Years	Males (mg/day)	Females (mg/day)	Age in Years	Males (mg/day)	Females (mg/day)
Children	1–3	0.7	0.7	1–3	3	3
	4–8	1	1	4–8	5	5
	9–13	2	2	9–13 years	8	8
Adolescents	14–18	3	3	14–18 years	11	9
Adults	≥19	4	3	≥19 years	11	9
Pregnant	≥19 years	-	3	≥19 years	-	11
Breastfeeding	≥19 years	-	3	≥19 years	-	12

Sources: Khayat et al. 2017 [8]; National Institute of Health, 2021; National Institute of Health, 2021.

**Table 4:** Recommended dietary allowance for manganese and copper.

	Manganese			Copper		
Life Stage	Age in Years	Males (mg/day)	Females (mg/day)	Age in Years	Males (µg/day)	Females (µg/day)
Children	1–3	1.2	1.2	1–3	340	340
	4–8	1.5	1.5	4–8	440	440
	9–13	1.9	1.6	9–13	700	700
Adolescents	14–18	2.2	1.6	14–18	890	890
Adults	≥19	2.3	1.8	≥19	900	900
Pregnant	≥19 years	-	2	≥19 years	-	1300
Breastfeeding	≥19 years	-	2.6	≥19 years	-	1300

Sources: Khayat et al. 2017 [8]; National Institute of Health, 2021; National Institute of Health, 2021.

## Abbreviations

ADHD	Attention Deficits and Hyperactive Disorder;
BMI	Body Mass Index;
BDNF	Brain-Derived Neurotropic Factor;
CAT	Catalase;
CNS	Central Nervous System;
Co	Cobalt;
EPA	Environmental Protection Agency;
FDA	Food and Drug Administration;
IDA	Iron deficiency anaemia;
IDD	Iodine deficiency disorder;
IQ	Intelligence Quotient;
LBW	Low birth weight;
MetS	Metabolic syndrome;
PIH	Pregnancy-induced hypertension syndrome;
PTB	Preterm birth;
PTB	Preterm birth;
UTI	Urinary Tract Infection;
RDA	Recommended Dietary Allowance;
WHO	World Health Organization;
Zfp	Zinc finger proteins

## References

[1] Zoroddu M.A., Aaseth J., Crisponi G., Medici S., Peana M., Nurchi V.M. (2019). The Essential Metals for Humans: A Brief Overview. J. Inorg. Biochem..

[2] Berdanier C., Dwyer D., Heber D. (2016). Hand Book of Nutrition and Food.

[3] Awuchi C.G.I., Ikechukwu V.S.A., Echeta C.K. (2020). Health Benefits of Micronutrients (Vitamins and Minerals) and Their Associated Deficiency Diseases: A Systematic Review. Int. J. Food Sci..

[4] Awuchi C., Victory I., Echeta C. (2019). The Functional Properties of Foods and Flours. Int. J. Adv. Acad. Res..

[5] Blancquaert D., Steur H.D., Gellynck X., Straeten D.V.D. (2017). Metabolic Engineering of Micronutrients in Crop Plants. Ann. N. Y. Acad. Sci..

[6] Gernand A.D., Schulze K.J., Stewart C.P., West K.P., Christian P. (2016). Micronutrient Deficiencies in Pregnancy Worldwide: Health Effects and Prevention. Nat. Rev. Endocrinol..

[7] Moran V.H. (2011). Minerals and Pregnancy.

[8] Khayat S., Fanaei H., Ghanbarzehi A. (2017). Minerals in Pregnancy and Lactation: A Review Article. J. Clin. Diagn. Res. JCDR.

[9] Liochev S.I. (2013). Reactive Oxygen Species and the Free Radical Theory of Aging. Free Radic. Biol. Med..

[10] Rodier F., Zhou D., Ferbeyre G. (2018). Cellular Senescence, Geroscience, Cancer and Beyond. Aging.

[11] Mravunac M., Szymlek-Gay E.A., Daly R.M., Roberts B.R., Formica M., Gianoudis J., O’Connell S.L., Nowson C.A., Cardoso B.R. (2019). Greater Circulating Copper Concentrations and Copper/Zinc Ratios Are Associated with Lower Psychological Distress, But Not Cognitive Performance, in a Sample of Australian Older Adults. Nutrients.

[12] Pfrimer K., Ferriolli E., Takeuchi P.L., Salles M.S., Saran-Netto A., Zanetti M.A., Roma-Junior L.C., Braga C.B.M., Domenici F.A., Valim Y.M. (2018). Effects of the Consumption of Milk Biofortified with Selenium, Vitamin E, and Different Fatty Acid Profile on Immune Response in the Elderly. Mol. Nutr. Food Res..

[13] Ponikowski P., van Veldhuisen D.J., Comin-Colet J., Ertl G., Komajda M., Mareev V., McDonagh T., Parkhomenko A., Tavazzi L., Levesque V. (2015). Beneficial Effects of Long-Term Intravenous Iron Therapy with Ferric Carboxymaltose in Patients with Symptomatic Heart Failure and Iron Deficiency. Eur. Heart J..

[14] Haryanto B., Suksmasari T., Wintergerst E., Maggini S. (2015). Multivitamin Supplementation Supports Immune Function and Ameliorates Conditions Triggered By Reduced Air Quality. Vitam. Miner..

[15] Kieliszek M., Błażejak S. (2013). Selenium: Significance, and Outlook for Supplementation. Nutrition.

[16] Saeed F., Nadeem M., Ahmed R.S., Nadeem M.T., Arshad M.S., Ullah A. (2016). Studying the Impact of Nutritional Immunology Underlying the Modulation of Immune Responses by Nutritional Compounds—A Review. Food Agric. Immunol..

[17] (2020). National Institute of Health Selenium Fact Sheet for Health Professionals. https://ods.od.nih.gov/Factsheets/Selenium-HealthProfessional/#en2.

[18] Terry E.N., Diamond A.M., Erdman J., Macdonald I., Zeisel S. (2012). Selenium. Present Knowledge in Nutrition.

[19] Galan-Chilet I., Tellez-Plaza M., Guallar E., de Marco G., Lopez-Izquierdo R., Gonzalez-Manzano I., Tormos M.C., Martin-Nuñez G.M., Rojo-Martinez G., Saez G.T. (2014). Plasma Selenium Levels and Oxidative Stress Biomarkers: A Gene-Environment Interaction Population-Based Study. Free Radic. Biol. Med..

[20] Duntas L.H., Benvenga S. (2015). Selenium: An Element for Life. Endocrine.

[21] Alahmadi B.A., El-Alfy S.H., Hemaid A.M., Abdel-Nabi I.M. (2020). The Protective Effects of Vitamin E against Selenium-Induced Oxidative Damage and Hepatotoxicity in Rats. J. Taibah Univ. Sci..

[22] Kieliszek M., Błażejak S. (2016). Current Knowledge on the Importance of Selenium in Food for Living Organisms: A Review. Molecules.

[23] Bampidis V., Azimonti G., de Lourdes Bastos M., Christensen H., Dusemund B., Kouba M., Kos Durjava M., López-Alonso M., López Puente S., Marcon F. (2019). Assessment of the Application for Renewal of Authorisation of Selenomethionine Produced by Saccharomyces Cerevisiae NCYC R397 for All Animal Species. EFSA J..

[24] Kipp A.P., Strohm D., Brigelius-Flohé R., Schomburg L., Bechthold A., Leschik-Bonnet E., Heseker H., German Nutrition Society (DGE) (2015). Revised Reference Values for Selenium Intake. J. Trace Elem. Med. Biol..

[25] Higdon J., Drake V., Delage B., Tsuji P. Selenium. https://lpi.oregonstate.edu/mic/minerals/selenium.

[26] Ross A.C., Caballero B.H., Cousins R.J., Tucker K.L., Ziegler T.R. (2012). Modern Nutrition in Health and Disease: Eleventh Edition.

[27] Wang L., Yin J., Yang B., Qu C., Lei J., Han J., Guo X. (2020). Serious Selenium Deficiency in the Serum of Patients with Kashin-Beck Disease and the Effect of Nano-Selenium on Their Chondrocytes. Biol. Trace Elem. Res..

[28] Hubalewska-Dydejczyk A., Duntas L., Gilis-Januszewska A. (2020). Pregnancy, thyroid, and the potential use of selenium. Hormones.

[29] Bizerea T.O., Dezsi S.G., Marginean O., Stroescu R., Rogobete A., Bizerea-Spiridon O., Ilie C. (2018). The Link Between Selenium, Oxidative Stress and Pregnancy Induced Hypertensive Disorders. Clin. Lab..

[30] Rayman M.P. (2012). Selenium and Human Health. Lancet Lond. Engl..

[31] Lewandowska M., Sajdak S., Lubiński J. (2019). Serum Selenium Level in Early Healthy Pregnancy as a Risk Marker of Pregnancy Induced Hypertension. Nutrients.

[32] Tindell R., Tipple T. (2018). Selenium: Implications for Outcomes in Extremely Preterm Infants. J. Perinatol..

[33] Nourbakhsh M., Ahmadpour F., Chahardoli B., Malekpour-Dehkordi Z., Nourbakhsh M., Hosseini-Fard S.R., Doustimotlagh A., Golestani A., Razzaghy-Azar M. (2016). Selenium and Its Relationship with Selenoprotein P and Glutathione Peroxidase in Children and Adolescents with Hashimoto’s Thyroiditis and Hypothyroidism. J. Trace Elem. Med. Biol..

[34] Cai Z., Zhang J., Li H. (2019). Selenium, Aging and Aging-Related Diseases. Aging Clin. Exp. Res..

[35] Jenzer H. (2016). Iodine: Biochemistry, Deficiency and Application in Clinical Nutrition. Can. J. Clin. Nutr..

[36] Pearce E.N. (2014). Iodine Deficiency in Children. Paediatr. Thyroidol..

[37] Zimmermann M.B. (2009). Iodine Deficiency. Endocr. Rev..

[38] Lazarus J.H. (2015). The Importance of Iodine in Public Health. Environ. Geochem. Health.

[39] Sun X., Shan Z., Teng W. (2014). Effects of Increased Iodine Intake on Thyroid Disorders. Endocrinol. Metab. Seoul Korea.

[40] Zimmermann M., Trumbo P.R. (2013). Iodine1. Adv. Nutr..

[41] National Institute of Health Office of Dietary Supplements—Iodine. https://ods.od.nih.gov/factsheets/Iodine-HealthProfessional/.

[42] Zimmermann M.B. (2016). The Importance of Adequate Iodine during Pregnancy and Infancy. Hidden Hunger.

[43] Melse-Boonstra A., Jaiswal N. (2010). Iodine Deficiency in Pregnancy, Infancy and Childhood and Its Consequences for Brain Development. Best Pract. Res. Clin. Endocrinol. Metab..

[44] Moog N.K., Entringer S., Heim C., Wadhwa P.D., Kathmann N., Buss C. (2017). Influence of Maternal Thyroid Hormones during Gestation on Fetal Brain Development. Neuroscience.

[45] Kim M.I., Feingold K.R., Anawalt B., Boyce A., Chrousos G., de Herder W.W., Dhatariya K., Dungan K., Grossman A., Hershman J.M., Hofland J. (2000). Hypothyroidism in Older Adults. Endotext.

[46] Díez J. (2003). Hyperthyroidism in Patients Older than 55 Years: An Analysis of the Etiology and Management. Gerontology.

[47] O’Kane S.M., Pourshahidi L.K., Mulhern M.S., Strain J., Mackle E.M., Koca D., Schomburg L., Hill S., O’Reilly J., Kmiotek D. (2018). Cow Milk Consumption Increases Iodine Status in Women of Childbearing Age in a Randomized Controlled Trial. J. Nutr..

[48] Elbon S.M., Johnson M.A., Fischer J.G. (1998). Milk Consumption in Older Americans. Am. J. Public Health.

[49] Kaur D., Rasane P., Singh J., Kaur S., Kumar V., Mahato D.K., Dey A., Dhawan K., Kumar S. (2019). Nutritional Interventions for Elderly and Considerations for the Development of Geriatric Foods. Curr. Aging Sci..

[50] Dev S., Babitt J.L. (2017). Overview of Iron Metabolism in Health and Disease. Hemodial. Int. Int. Symp. Home Hemodial..

[51] (2014). Nutrient Reference Values—For Australia and New Zealand Iron.

[52] National Institute of Health Office of Dietary Supplements—Iron. https://ods.od.nih.gov/factsheets/Iron-HealthProfessional/.

[53] Nguyen P.H., Young M., Gonzalez-Casanova I., Pham H.Q., Nguyen H., Truong T.V., Nguyen S.V., Harding K.B., Reinhart G.A., Martorell R. (2016). Impact of Preconception Micronutrient Supplementation on Anemia and Iron Status during Pregnancy and Postpartum: A Randomized Controlled Trial in Rural Vietnam. PLoS ONE.

[54] Darling A.M., Mitchell A.A., Werler M.M. (2016). Preconceptional Iron Intake and Gestational Diabetes Mellitus. Int. J. Environ. Res. Public. Health.

[55] Behboudi-Gandevani S., Safary K., Moghaddam-Banaem L., Lamyian M., Goshtasebi A., Goshtasbi A., Alian-Moghaddam N. (2013). The Relationship between Maternal Serum Iron and Zinc Levels and Their Nutritional Intakes in Early Pregnancy with Gestational Diabetes. Biol. Trace Elem. Res..

[56] Helin A., Kinnunen T.I., Raitanen J., Ahonen S., Virtanen S.M., Luoto R. (2012). Iron Intake, Haemoglobin and Risk of Gestational Diabetes: A Prospective Cohort Study. BMJ Open.

[57] Guo Y., Zhang N., Zhang D., Ren Q., Ganz T., Liu S., Nemeth E. (2019). Iron Homeostasis in Pregnancy and Spontaneous Abortion. Am. J. Hematol..

[58] Jorgensen J.M., Yang Z., Lönnerdal B., Chantry C.J., Dewey K.G. (2017). Effect of Iron Supplementation during Lactation on Maternal Iron Status and Oxidative Stress: A Randomized Controlled Trial. Matern. Child. Nutr..

[59] Marin G., Mestorino N., Errecalde J., Huber B., Uriarte A., Orchuela J. (2012). Personalised Iron Supply for Prophylaxis and Treatment of Pregnant Women as a Way to Ensure Normal Iron Levels in Their Breast Milk. J. Med. Life.

[60] Friel J., Qasem W., Cai C. (2018). Iron and the Breastfed Infant. Antioxidants.

[61] Krebs N.F., Sherlock L.G., Westcott J., Culbertson D., Hambidge K.M., Feazel L.M., Robertson C.E., Frank D.N. (2013). Effects of Different Complementary Feeding Regimens on Iron Status and Enteric Microbiota in Breastfed Infants. J. Pediatr..

[62] Tang M., Frank D., Sherlock L., Ir D., Robertson C., Krebs N. (2016). Effect of Vitamin E With Therapeutic Iron Supplementation on Iron Repletion and Gut Microbiome in US Iron Deficient Infants and Toddlers. J. Pediatr. Gastroenterol. Nutr..

[63] Angulo-Barroso R.M., Li M., Santos D.C.C., Bian Y., Sturza J., Jiang Y., Kaciroti N., Richards B., Lozoff B. (2016). Iron Supplementation in Pregnancy or Infancy and Motor Development: A Randomized Controlled Trial. Pediatrics.

[64] Doom J.R., Richards B., Caballero G., Delva J., Gahagan S., Lozoff B. (2018). Infant Iron Deficiency and Iron Supplementation Predict Adolescent Internalizing, Externalizing, and Social Problems. J. Pediatr..

[65] Lange K.W., Hauser J., Lange K.M., Makulska-Gertruda E., Nakamura Y., Reissmann A., Sakaue Y., Takano T., Takeuchi Y. (2017). The Role of Nutritional Supplements in the Treatment of ADHD: What the Evidence Says. Curr. Psychiatry Rep..

[66] Chao K.-C., Chang C.-C., Chiou H.-Y., Chang J.-S. (2015). Serum Ferritin Is Inversely Correlated with Testosterone in Boys and Young Male Adolescents: A Cross-Sectional Study in Taiwan. PLoS ONE.

[67] Dhindsa S., Ghanim H., Batra M., Kuhadiya N.D., Abuaysheh S., Green K., Makdissi A., Chaudhuri A., Dandona P. (2016). Effect of Testosterone on Hepcidin, Ferroportin, Ferritin and Iron Binding Capacity in Patients with Hypogonadotropic Hypogonadism and Type 2 Diabetes. Clin. Endocrinol..

[68] Beggs L.A., Yarrow J.F., Conover C.F., Meuleman J.R., Beck D.T., Morrow M., Zou B., Shuster J.J., Borst S.E. (2014). Testosterone Alters Iron Metabolism and Stimulates Red Blood Cell Production Independently of Dihydrotestosterone. Am. J. Physiol. Endocrinol. Metab..

[69] El Osta R., Grandpre N., Monnin N., Hubert J., Koscinski I. (2017). Hypogonadotropic Hypogonadism in Men with Hereditary Hemochromatosis. Basic Clin. Androl..

[70] von H.S., Ebner N., Evertz R., Ponikowski P., Anker S.D. (2019). Iron Deficiency in Heart Failure. JACC Heart Fail..

[71] Portolés J., Martín L., Broseta J.J., Cases A. (2021). Anemia in Chronic Kidney Disease: From Pathophysiology and Current Treatments, to Future Agents. Front. Med..

[72] Lewis J.B., Sika M., Koury M.J., Chuang P., Schulman G., Smith M.T., Whittier F.C., Linfert D.R., Galphin C.M., Athreya B.P. (2015). Ferric Citrate Controls Phosphorus and Delivers Iron in Patients on Dialysis. J. Am. Soc. Nephrol. JASN.

[73] Hokin B., Adams M., Ashton J., Louie H. (2004). Comparison of the Dietary Cobalt Intake in Three Different Australian Diets. Asia Pac. J. Clin. Nutr..

[74] Adolfo F.R., Do Nascimento P.C., Bohrer D., De Carvalho L.M., Viana C., Guarda A., Nunes Colim A., Mattiazzi P. (2016). Simultaneous Determination of Cobalt and Nickel in Vitamin B12 Samples Using High-Resolution Continuum Source Atomic Absorption Spectrometry. Talanta.

[75] Tvermoes B.E., Finley B.L., Unice K.M., Otani J.M., Paustenbach D.J., Galbraith D.A. (2013). Cobalt Whole Blood Concentrations in Healthy Adult Male Volunteers Following Two-Weeks of Ingesting a Cobalt Supplement. Food Chem. Toxicol..

[76] National Institute of Health Office of Dietary Supplements—Vitamin B12. https://ods.od.nih.gov/factsheets/VitaminB12-HealthProfessional/.

[77] Marzec-Wróblewska U., Kamiński P., Łakota P., Szymański M., Wasilow K., Ludwikowski G., Jerzak L., Stuczyński T., Woźniak A., Buciński A. (2019). Human Sperm Characteristics with Regard to Cobalt, Chromium, and Lead in Semen and Activity of Catalase in Seminal Plasma. Biol. Trace Elem. Res..

[78] Li Z.-J., Liang C.-M., Xia X., Huang K., Yan S.-Q., Tao R.W., Pan W.-J., Sheng J., Tao Y.-R., Xiang H.-Y. (2019). Association between Maternal and Umbilical Cord Serum Cobalt Concentration during Pregnancy and the Risk of Preterm Birth: The Ma’anshan Birth Cohort (MABC) Study. Chemosphere.

[79] Angelova M.G., Petkova-Marinova T.V., Pogorielov M.V., Loboda A.N., Nedkova-Kolarova V.N., Bozhinova A.N. (2014). Trace Element Status (Iron, Zinc, Copper, Chromium, Cobalt, and Nickel) in Iron-Deficiency Anaemia of Children under 3 Years. Anemia.

[80] Kumar N., Sood S., Arora B., Singh M., Beena (2010). Effect of Duration of Fluoride Exposure on the Reproductive System in Male Rabbits. J. Hum. Reprod. Sci..

[81] Aldrees A., Al-Manea S. (2010). Fluoride Content of Bottled Drinking Waters Available in Riyadh, Saudi Arabia. Saudi Dent. J..

[82] Aoun A., Darwiche F., Al Hayek S., Doumit J. (2018). The Fluoride Debate: The Pros and Cons of Fluoridation. Prev. Nutr. Food Sci..

[83] National Institute of Health Office of Dietary Supplements—Fluoride. https://ods.od.nih.gov/factsheets/Fluoride-HealthProfessional/.

[84] Chaithra B., Sarjan H.N., Shivabasavaiah (2020). A Comparative Analysis of Fluoride-Contaminated Groundwater and Sodium Fluoride-Induced Reproductive Toxicity and Its Reversibility in Male Rats. Biol. Trace Elem. Res..

[85] Darmani H., Al-Hiyasat A.S., Irbid A.M.E. (2001). Effects of Sodium Fluoride in Drinking Water on Fertility in Female Mice. Fluoride.

[86] Abduweli Uyghurturk D., Goin D.E., Martinez-Mier E.A., Woodruff T.J., DenBesten P.K. (2020). Maternal and Fetal Exposures to Fluoride during Mid-Gestation among Pregnant Women in Northern California. Environ. Health.

[87] Green R., Lanphear B., Hornung R., Flora D., Martinez-Mier E.A., Neufeld R., Ayotte P., Muckle G., Till C. (2019). Association Between Maternal Fluoride Exposure During Pregnancy and IQ Scores in Offspring in Canada. JAMA Pediatr..

[88] Goyal L.D., Bakshi D.K., Arora J.K., Manchanda A., Singh P. (2020). Assessment of Fluoride Levels during Pregnancy and Its Association with Early Adverse Pregnancy Outcomes. J. Fam. Med. Prim. Care.

[89] Shivarajashankara Y., Shivashankara A., Rao S.H., Karnataka P.G.B. (2001). Oxidative Stress in Children with Endemic Skeletal Fluorosis. Fluoride.

[90] Liu Y., Téllez-Rojo M., Hu H., Sánchez B.N., Martinez-Mier E.A., Basu N., Mercado-García A., Solano-González M., Peterson K.E. (2019). Fluoride Exposure and Pubertal Development in Children Living in Mexico City. Environ. Health.

[91] Malin A.J., Bose S., Busgang S.A., Gennings C., Thorpy M., Wright R.O., Wright R.J., Arora M. (2019). Fluoride Exposure and Sleep Patterns among Older Adolescents in the United States: A Cross-Sectional Study of NHANES 2015–2016. Environ. Health.

[92] Toumba K.J., Twetman S., Splieth C., Parnell C., van Loveren C., Lygidakis N.A. (2019). Guidelines on the Use of Fluoride for Caries Prevention in Children: An Updated EAPD Policy Document. Eur. Arch. Paediatr. Dent. Off. J. Eur. Acad. Paediatr. Dent..

[93] Petersen P.E. (2016). Editorial - Prevention of Dental Caries through the Use of FLuoride—The WHO Approach. Community Dent. Health.

[94] O’Mullane D.M. (2016). Fluoride and Oral Health. Community Dent. Health.

[95] Griffin S.O., Regnier E., Griffin P.M., Huntley V. (2007). Effectiveness of Fluoride in Preventing Caries in Adults. J. Dent. Res..

[96] Souza-Rodrigues R.D., da Silva Ferreira S., D’almeida-Couto R.S., Lachowski K.M., Sobral M.Â.P., Marques M.M. (2015). Choice of Toothpaste for the Elderly: An *in Vitro* Study. Braz. Oral Res..

[97] Ekstrand K.R. (2016). High Fluoride Dentifrices for Elderly and Vulnerable Adults: Does It Work and If So, Then Why?. Caries Res..

[98] Chasapis C.T., Spiliopoulou C.A., Loutsidou A.C., Stefanidou M.E. (2012). Zinc and Human Health: An Update. Arch. Toxicol..

[99] National Institute of Health Office of Dietary Supplements—Zinc. https://ods.od.nih.gov/factsheets/Zinc-HealthProfessional/.

[100] Kerns K., Zigo M., Sutovsky P. (2018). Zinc: A Necessary Ion for Mammalian Sperm Fertilization Competency. Int. J. Mol. Sci..

[101] Fallah A., Mohammad-Hasani A., Colagar A.H. (2018). Zinc Is an Essential Element for Male Fertility: A Review of Zn Roles in Men’s Health, Germination, Sperm Quality, and Fertilization. J. Reprod. Infertil..

[102] Sørensen M.B., Bergdahl I.A., Hjøllund N.H.I., Bonde J.P.E., Stoltenberg M., Ernst E. (1999). Zinc, Magnesium and Calcium in Human Seminal Fluid: Relations to Other Semen Parameters and Fertility. Mol. Hum. Reprod..

[103] Alemu B., Gashu D. (2020). Association of Maternal Anthropometry, Hemoglobin and Serum Zinc Concentration during Pregnancy with Birth Weight. Early Hum. Dev..

[104] Bhutta Z.A., Black R.E., Brown K.H., Meeks Gardner J., Gore S., Hidayat A., Khatun F., Martorell R., Ninb N.X., Penny M.E. (1999). Prevention of Diarrhea and Pneumonia by Zinc Supplementation in Children in Developing Countries: Pooled Analysis of Randomized Controlled Trials. J. Pediatr..

[105] Kaur K., Gupta R., Saraf S.A., Saraf S.K. (2014). Zinc: The Metal of Life. Compr. Rev. Food Sci. Food Saf..

[106] Valavi E., Hakimzadeh M., Shamsizadeh A., Aminzadeh M., Alghasi A. (2011). The Efficacy of Zinc Supplementation on Outcome of Children with Severe Pneumonia. A Randomized Double-Blind Placebo-Controlled Clinical Trial. Indian J. Pediatr..

[107] Basnet S., Shrestha P.S., Sharma A., Mathisen M., Prasai R., Bhandari N., Adhikari R.K., Sommerfelt H., Valentiner-Branth P., Strand T.A. (2012). A Randomized Controlled Trial of Zinc as Adjuvant Therapy for Severe Pneumonia in Young Children. Pediatrics.

[108] Sempértegui F., Estrella B., Rodríguez O., Gómez D., Cabezas M., Salgado G., Sabin L.L., Hamer D.H. (2014). Zinc as an Adjunct to the Treatment of Severe Pneumonia in Ecuadorian Children: A Randomized Controlled Trial. Am. J. Clin. Nutr..

[109] Shah G.S., Dutta A.K., Shah D., Mishra O.P. (2012). Role of Zinc in Severe Pneumonia: A Randomized Double Bind Placebo Controlled Study. Ital. J. Pediatr..

[110] Zabihi F., Mostafavi M., Esmaeili M., Cheshani M.I. (2020). Investigating the Effect of Zinc Deficiency on the Risk of Urinary Tract Infection in Children. Int. J. Pediatr..

[111] Negri A.L. (2018). The Role of Zinc in Urinary Stone Disease. Int. Urol. Nephrol..

[112] Sun Y., Wang Y., Wang D., Zhou Q. (2020). Dietary Zinc Intake, Supplemental Zinc Intake and Serum Zinc Levels and the Prevalence of Kidney Stones in Adults. J. Trace Elem. Med. Biol..

[113] Perrotta F., Corbi G., Mazzeo G., Boccia M., Aronne L., D’Agnano V., Komici K., Mazzarella G., Parrella R., Bianco A. (2020). COVID-19 and the Elderly: Insights into Pathogenesis and Clinical Decision-Making. Aging Clin. Exp. Res..

[114] Amore S., Puppo E., Melara J., Terracciano E., Gentili S., Liotta G. (2021). Impact of COVID-19 on Older Adults and Role of Long-Term Care Facilities during Early Stages of Epidemic in Italy. Sci. Rep..

[115] de Almeida Brasiel P.G. (2020). The Key Role of Zinc in Elderly Immunity: A Possible Approach in the COVID-19 Crisis. Clin. Nutr. ESPEN.

[116] Fang C., Wu W., Gu X., Dai S., Zhou Q., Deng H., Shen F., Chen J. (2019). Association of Serum Copper, Zinc and Selenium Levels with Risk of Metabolic Syndrome: A Nested Case-Control Study of Middle-Aged and Older Chinese Adults. J. Trace Elem. Med. Biol..

[117] Yeung D.C.Y., Lam K.S.L., Wang Y., Tso A.W.K., Xu A. (2009). Serum Zinc-A2-Glycoprotein Correlates with Adiposity, Triglycerides, and the Key Components of the Metabolic Syndrome in Chinese Subjects. J. Clin. Endocrinol. Metab..

[118] Ghasemi A., Zahediasl S., Hosseini-Esfahani F., Azizi F. (2014). Gender Differences in the Relationship between Serum Zinc Concentration and Metabolic Syndrome. Ann. Hum. Biol..

[119] Chen P., Bornhorst J., Aschner M. (2018). Manganese Metabolism in Humans. Front. Biosci. Landmark Ed..

[120] Kumar K.K., Lowe J., Aboud A.A., Neely M.D., Redha R., Bauer J.A., Odak M., Weaver C.D., Meiler J., Aschner M. (2014). Cellular Manganese Content Is Developmentally Regulated in Human Dopaminergic Neurons. Sci. Rep..

[121] Lucchini R.G., Aschner M., Kim Y., Šarić M. (2015). Manganese. Handbook on the Toxicology of Metals.

[122] National Institute of Health Office of Dietary Supplements—Manganese. https://ods.od.nih.gov/factsheets/Manganese-HealthProfessional/.

[123] McRae N., Bello G., Svensson K., Solano-González M., Wright R.J., Niedzwiecki M.M., Calapiz M.T., Amarasiriwardena C., Schnaas L., Tamayo-Ortiz M. (2020). Blood Manganese Levels during Pregnancy and Postpartum Depression: A Cohort Study among Women in Mexico. Neurotoxicology.

[124] Gromova O.A., Andreeva E.N., Torshin I.Y., Tapilskaya N.I., Uvarova E.V. (2020). A systemic biological analysis of the role of manganese in obstetrics and gynaecology: Women’s reproductive health, menstrual cycle regulation and prevention of fetal malformations. Gynecol. Obstet. Perinatol..

[125] Slicker J., Vermilyea S. (2009). Pediatric Parenteral Nutrition: Putting the Microscope on Macronutrients and Micronutrients. Nutr. Clin. Pract..

[126] Erikson K.M., Aschner M., Carver P.L. (2019). Manganese: Its Role in Disease and Health. Essential Metals in Medicine: Therapeutic Use and Toxicity of Metal Ions in the Clinic.

[127] Bailey R.L., Fulgoni V.L., Keast D.R., Dwyer J.T. (2011). Dietary Supplement Use Is Associated with Higher Intakes of Minerals from Food Sources. Am. J. Clin. Nutr..

[128] Stierman B., Woolf A., Korrick S. (2019). Associations of Prenatal Manganese with Visual-Motor Skills in Adolescence. Clin. Toxicol..

[129] Gong J.H., Lo K., Liu Q., Li J., Lai S., Shadyab A.H., Arcan C., Snetselaar L., Liu S. (2020). Dietary Manganese, Plasma Markers of Inflammation, and the Development of Type 2 Diabetes in Postmenopausal Women: Findings From the Women’s Health Initiative. Diabetes Care.

[130] Kresovich J.K., Bulka C.M., Joyce B.T., Vokonas P.S., Schwartz J., Baccarelli A.A., Hibler E.A., Hou L. (2018). The Inflammatory Potential of Dietary Manganese in a Cohort of Elderly Men. Biol. Trace Elem. Res..

[131] Eaton-Evans J., Mcllrath E.M., Jackson W.E., McCartney H., Strain J.J. (1996). Copper Supplementation and the Maintenance of Bone Mineral Density in Middle-Aged Women. J. Trace Elem. Exp. Med..

[132] Al-khateeb E., Al-zayadneh E., Al-dalahmah O., Alawadi Z., khatib F., Naffa R., Shafagoj Y. (2014). Relation between Copper, Lipid Profile, and Cognition in Elderly Jordanians. J. Alzheimers Dis. JAD.

[133] National Institute of Health Office of Dietary Supplements—Copper. https://ods.od.nih.gov/factsheets/Copper-HealthProfessional/.

[134] Kashanian M., Hadizadeh H., Faghankhani M., Nazemi M., Sheikhansari N. (2018). Evaluating the Effects of Copper Supplement during Pregnancy on Premature Rupture of Membranes and Pregnancy Outcome. J. Matern.-Fetal Neonatal Med..

[135] Sak S., Barut M., Çelik H., Incebiyik A., Ağaçayak E., Uyanikoglu H., Kirmit A., Sak M. (2020). Copper and Ceruloplasmin Levels Are Closely Related to the Severity of Preeclampsia. J. Matern.-Fetal Neonatal Med..

[136] Latorre M., Troncoso R., Uauy R., Kerkar N., Roberts E.A. (2019). Chapter 4 - Biological Aspects of Copper. Clinical and Translational Perspectives on WILSON DISEASE.

[137] Olivares M., Lönnerdal B., Abrams S.A., Pizarro F., Uauy R. (2002). Age and Copper Intake Do Not Affect Copper Absorption, Measured with the Use of 65Cu as a Tracer, in Young Infants. Am. J. Clin. Nutr..

[138] Domellof M., Koletzko B., Poindexter B., Uauy R. (2014). Nutritional Care of Premature Infants: Microminerals. Nutritional Care of Preterm Infants: Scientific Basis and Practical Guidelines.

[139] Nakamura M., Miura A., Nagahata T., Shibata Y., Okada E., Ojima T. (2019). Low Zinc, Copper, and Manganese Intake Is Associated with Depression and Anxiety Symptoms in the Japanese Working Population: Findings from the Eating Habit and Well-Being Study. Nutrients.

[140] Ni M., You Y., Chen J., Zhang L. (2018). Copper in Depressive Disorder: A Systematic Review and Meta-Analysis of Observational Studies. Psychiatry Res..

[141] Styczeń K., Sowa-Kućma M., Siwek M., Dudek D., Reczyński W., Misztak P., Szewczyk B., Topór-Mądry R., Opoka W., Nowak G. (2016). Study of the Serum Copper Levels in Patients with Major Depressive Disorder. Biol. Trace Elem. Res..

[142] Sfar S., Jawed A., Braham H., Amor S., Laporte F., Kerkeni A. (2009). Zinc, Copper and Antioxidant Enzyme Activities in Healthy Elderly Tunisian Subjects. Exp. Gerontol..

[143] Tsuboi A., Terazawa-Watanabe M., Kazumi T., Fukuo K. (2015). Associations of Decreased Serum Transthyretin with Elevated High-Sensitivity CRP, Serum Copper and Decreased Hemoglobin in Ambulatory Elderly Women. Asia Pac. J. Clin. Nutr..

[144] Tsongas T.A., Meglen R.R., Walravens P.A., Chappell W.R. (1980). Molybdenum in the Diet: An Estimate of Average Daily Intake in the United States. Am. J. Clin. Nutr..

[145] Novotny J.A. (2011). Molybdenum Nutriture in Humans. J. Evid.-Based Complement. Altern. Med..

[146] Garrett R.M., Johnson J.L., Graf T.N., Feigenbaum A., Rajagopalan K.V. (1998). Human Sulfite Oxidase R160Q: Identification of the Mutation in a Sulfite Oxidase-Deficient Patient and Expression and Characterization of the Mutant Enzyme. Proc. Natl. Acad. Sci. USA.

[147] Wahl B., Reichmann D., Niks D., Krompholz N., Havemeyer A., Clement B., Messerschmidt T., Rothkegel M., Biester H., Hille R. (2010). Biochemical and Spectroscopic Characterization of the Human Mitochondrial Amidoxime Reducing Components HmARC-1 and HmARC-2 Suggests the Existence of a New Molybdenum Enzyme Family in Eukaryotes. J. Biol. Chem..

[148] World Health Organization, International Atomic Energy Agency, Food and Agriculture Organization of the United Nations (1996). Trace Elements in Human Nutrition and Health.

[149] Reiss J., Hahnewald R. (2011). Molybdenum Cofactor Deficiency: Mutations in GPHN, MOCS1, and MOCS2. Hum. Mutat..

[150] Novotny J.A., Turnlund J.R. (2007). Molybdenum Intake Influences Molybdenum Kinetics in Men. J. Nutr..

[151] Vázquez-Salas R.A., López-Carrillo L., Menezes-Filho J.A., Rothenberg S.J., Cebrián M.E., Schnaas L., de Souza Viana G.F., Torres-Sánchez L. (2014). Prenatal Molybdenum Exposure and Infant Neurodevelopment in Mexican Children. Nutr. Neurosci..

[152] Zheng Y., Zhang C., Weisskopf M., Williams P.L., Parsons P.J., Palmer C.D., Buck Louis G.M., James-Todd T. (2019). A Prospective Study of Early Pregnancy Essential Metal(Loid)s and Glucose Levels Late in the Second Trimester. J. Clin. Endocrinol. Metab..

[153] Lee E.J., Dandamudi R., Granadillo J.L., Grange D.K., Kakajiwala A. (2021). Rare Cause of Xanthinuria: A Pediatric Case of Molybdenum Cofactor Deficiency B. CEN Case Rep..

[154] Schuierer G., Kurlemann G., Bick U., Stephani U. (1995). Molybdenum-Cofactor Deficiency: CT and MR Findings. Neuropediatrics.

[155] Menke A., Guallar E., Cowie C.C. (2016). Metals in Urine and Diabetes in U.S. Adults. Diabetes.

[156] Pandey R., Singh S.P. (2002). Effects of Molybdenum on Fertility of Male Rats. Biometals.

[157] Zhai X.-W., Zhang Y.-L., Qi Q., Bai Y., Chen X.-L., Jin L.-J., Ma X.-G., Shu R.-Z., Yang Z.-J., Liu F.-J. (2013). Effects of Molybdenum on Sperm Quality and Testis Oxidative Stress. Syst. Biol. Reprod. Med..

[158] Meeker J.D., Rossano M.G., Protas B., Padmanabhan V., Diamond M.P., Puscheck E., Daly D., Paneth N., Wirth J.J. (2010). Environmental Exposure to Metals and Male Reproductive Hormones: Circulating Testosterone Is Inversely Associated with Blood Molybdenum. Fertil. Steril..

